# TRIM21 is critical for survival of *Toxoplasma gondii* infection and localises to GBP-positive *parasite* vacuoles

**DOI:** 10.1038/s41598-017-05487-7

**Published:** 2017-07-12

**Authors:** Clémence Foltz, Anna Napolitano, Rabia Khan, Barbara Clough, Elizabeth M. Hirst, Eva-Maria Frickel

**Affiliations:** The Francis Crick Institute, 1 Midland Road, London, NW1 1AT UK

## Abstract

Interferon gamma (IFNγ) is the major proinflammatory cytokine conferring resistance to the intracellular vacuolar pathogen *Toxoplasma gondii* by inducing the destruction of the parasitophorous vacuole (PV). We previously identified TRIM21 as an IFNγ-driven E3 ubiquitin ligase mediating the deposition of ubiquitin around pathogen inclusions. Here, we show that TRIM21 knockout mice were highly susceptible to *Toxoplasma* infection, exhibiting decreased levels of serum inflammatory cytokines and higher parasite burden in the peritoneum and brain. We demonstrate that IFNγ drives recruitment of TRIM21 to GBP1-positive *Toxoplasma* vacuoles, leading to Lys63-linked ubiquitination of the vacuole and restriction of parasite early replication without interfering with vacuolar disruption. As seen *in vivo*, TRIM21 impacted the secretion of inflammatory cytokines. This study identifies TRIM21 as a previously unknown modulator of *Toxoplasma gondii* resistance *in vivo* thereby extending host innate immune recognition of eukaryotic pathogens to include E3 ubiquitin ligases.

## Introduction

Toxoplasmosis is an infectious disease that is caused by an obligate intracellular parasite belonging to the phylum Apicomplexa called *Toxoplasma gondii*. This unicellular parasite is found throughout the world and is arguably the most successful parasite with a global human infection rate of about 30%^1^. *Toxoplasma* is an opportunistic parasite causing chronic infection in humans that remains asymptomatic in many cases. However, complications for the foetus such as abortion, mental abnormalities or ocular disease can develop if an acute infection is acquired during pregnancy. Also, immunocompromised individuals can develop toxoplasmic encephalitis or retinochorioditis^2^ with the latter being a risk even for immunocompetent adults.

*Toxoplasma* can infect any nucleated cell in any warm-blooded animal, including humans. Inside the host cell, *Toxoplasma* resides and replicates within a parasitophorous vacuole (PV) formed during invasion by invagination of the host plasma membrane^3^. The majority of lipids composing the PV membrane (PVM) are host cell derived, and *Toxoplasma* regulates the contents of the PVM by preventing host proteins like SNAREs to accumulate at the PVM^4^. Thus, by never fusing with endo-lysosomes^5^ and being resistant to acidification^6^, the PV provides a safe and protective place for the parasite to survive in the host cell, allowing it to persist despite a vigorous immune response.

Type II interferon γ (IFNγ) was identified as the major proinflammatory cytokine that confers resistance against *Toxoplasma* ^7, 8^. Upon *Toxoplasma* infection, IFNγ mediates the deployment of a range of host defence molecules to the PV, ultimately leading to its disruption, autophagic elimination and inflammasome activation^9^. Central players in this defence mechanism are immunity-related GTPases (IRGs)^10–13^ and guanylate binding proteins (GBPs)^14^. These large GTPases recognise vacuoles of intracellular pathogens for destruction and clearance, as well as govern the subsequent activation of the inflammasome^15–21^. We have recently shown that ubiquitin is another central player in the IFNγ-dependent vacuolar recognition cascade in both mouse^22^ and human cells^23^. Ubiquitin is recruited to type II and III (Pru and CEP) *Toxoplasma* vacuoles in dependence of IRG proteins and the E3 ligase tumor necrosis factor (TNF) receptor associated factor 6 (TRAF6). Removal of the IFNγ-inducible ubiquitination pathway also substantially diminishes the p62-dependent delivery of GBPs to PVs and thus diminishes the host’s ability to restrict *Toxoplasma* replication^22^. Ubiquitin undoubtedly serves as a host-induced pattern that marks intracellular structures as immune targets for members of the GBP family of host defense proteins.

Ubiquitin deposition around a pathogen had already been well-established as a central dogma to intracellular defence against bacterial pathogens^24, 25^. The E3 ubiquitin ligase LRSAM1 has been shown to directly recognise *Salmonella*, leading to ubiquitination associated with the bacterium. LRSAM1 also participates in the recruitment of LC3-positive autophagosome by binding to the autophagy receptor nuclear dot protein 52 (NDP52)^26^. The E3 ubiquitin ligase HOIL-1 is a component of the linear ubiquitination chain assembly complex (LUBAC)^27^. HOIL-1 knockout mice are highly susceptible to *Citrobacter rodentium* and *Toxoplasma gondii* infection, exhibiting unchecked parasite burden^28^. The E3 ubiquitin ligase Parkin is involved in mitophagy and confers susceptibility to Parkinson’s disease^29–32^. Mice deficient in Parkin succumb during *Mycobacterium tuberculosis* infection concurrent with a higher bacterial load^33^. Moreover, some E3 ubiquitin ligases act on both cell-autonomous restriction and immune response regulation during bacterial infection. *Listeria monocytogenes* infection is fatal in HOIL-1 knockout mice, that cannot control bacterial replication and present an impaired production of protective cytokines by macrophages^28^. The tripartite motif protein 21 (TRIM21) has been reported to bind to invading antibody-coated adenoviruses as well as *Salmonella typhimurium* in the cytosol, and target the virus to degradation by the proteasome by virtue of its E3 ligase activity^34–36^. Following adenovirus and *Salmonella* infection in mouse embryonic fibroblasts, TRIM21 was suggested to mediate the formation of Lys63-linked chains and upregulate IRF3, IRF5, IRF7, NF-κB and AP-1, thereby inducing the production of proinflammatory cytokines^35^. Recently, TRIM21 has also been reported to mediate recognition of viral RNA and DNA by the host sensors RIG-I and cGAS, respectively^37^.

We previously identified TRIM21 as an E3 ligase involved in the deposition of ubiquitin around *Chlamydia* inclusions^22^. However, the biological relevance of TRIM21 has only been studied in the context of viral infection *in vivo* and its role in resistance to bacteria or other pathogens remains unclear^37, 38^. Here, we demonstrate that TRIM21 knockout mice were highly susceptible to *Toxoplasma* infection and exhibited decreased levels of proinflammatory cytokines in their serum associated with higher parasite burden in the brain. TRIM21 deficiency led to an enhanced early replication of *Toxoplasma* without the disrupted vacuole displaying overt morphological differences compared to the wild-type vacuole. We show TRIM21 and GBP1 are being co-recruited to PVs of type II and III (Pru and CEP), but not PruRop16I and CEPRop18I *Toxoplasma*. Type II and III (Pru and CEP), but not CEPRop18I *Toxoplasma*, were decorated with ubiquitin in an IFNγ- and TRIM21-dependent manner, with TRIM21 partly mediating Lys63 ubiquitin linkages. Concurrent with our *in vivo* findings, TRIM21 deficiency led to an enhanced early replication of type II parasites and a disregulated production of proinflammatory cytokines. We define TRIM21 as a novel, crucial intracellular restriction factor during acute *Toxoplasma* infection acting on both the regulation of innate immune response to the parasite and the restriction of parasite replication.

## Results

### TRIM21 deficiency increases susceptibility to *Toxoplasma* infection *in vivo*

**Figure 1:**
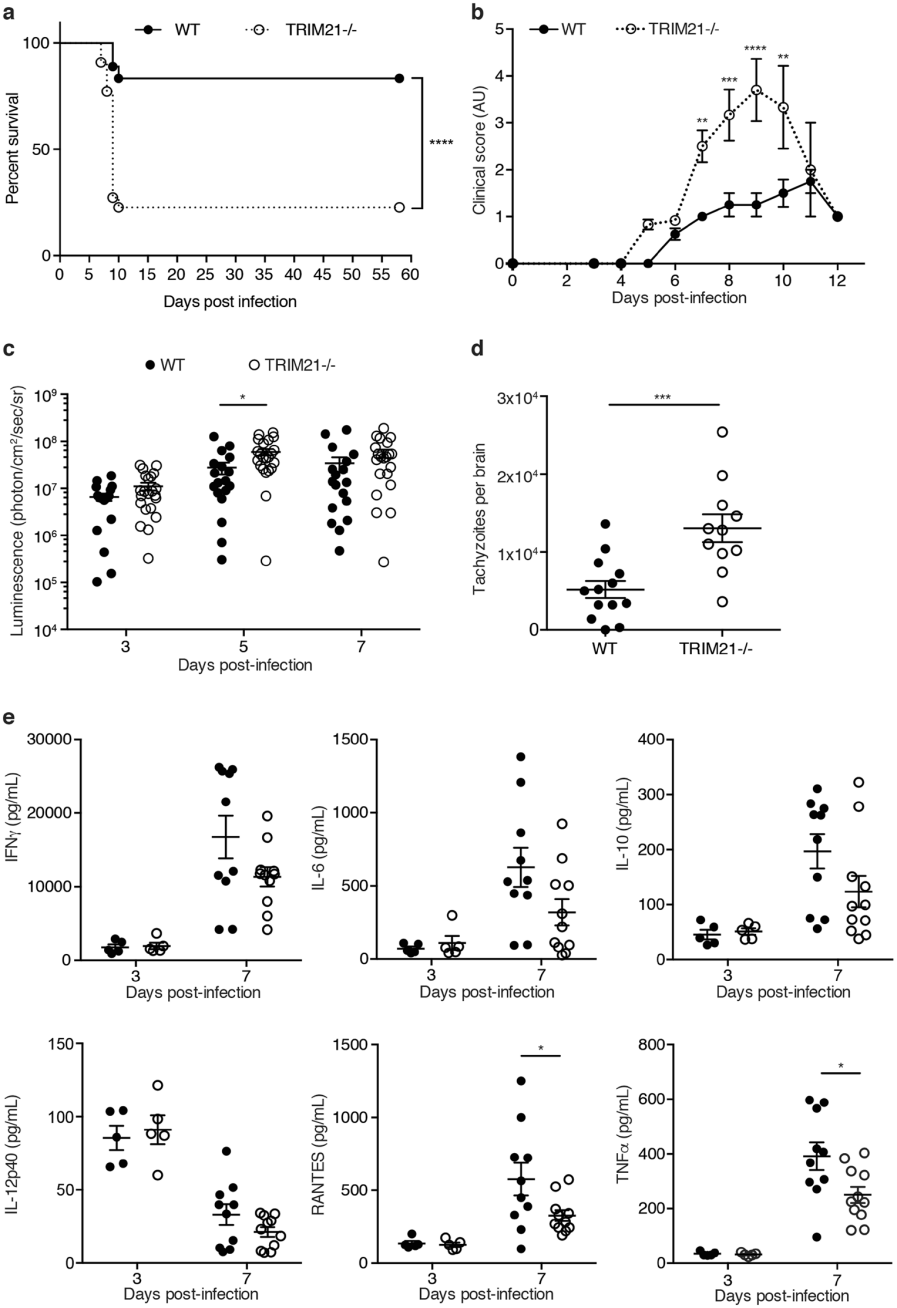
TRIM21-deficient mice are susceptible to *Toxoplasma* infection correlating with higher parasite burden and decreased levels of proinflammatory cytokines. (**a**) Survival curves of wild-type versus TRIM21-deficient mice infected intraperitoneally with 5 × 10^4^ type II *Toxoplasma* (n = 18 wild-type, n = 22 TRIM21 knockout). Curves were compared by log-rank survival analysis of Kaplan-Meier curves, ****p < 0.0001. (**b**) The clinical score, assessed by piloerection, a hunched position and immobility, was recorded throughout the time of the experiment (n = 18 wild-type, n = 22 TRIM21 knockout). Mean ± SEM, **p < 0.01, ***p < 0.001, ****p < 0.0001, 2-way ANOVA. (**c**) The evolution of the firefly luciferase-expressing parasite load in wild-type versus TRIM21-deficient mice was assessed by *in vivo* imaging after injection of luciferin at 3, 5 and 7 days post-infection (n = 18 wild-type, n = 22 TRIM21 knockout). Mean ± SEM, *p < 0.05, 2-way ANOVA. (**d**) Quantitative analysis of the number of GFP-expressing Toxoplasma tachyzoites in the brain of wild-type and TRIM21-deficient mice at 7d p.i. (n = 14 wild-type, n = 11 TRIM21 knockout). Data pooled from three independent experiments. Mean ± SEM, ***p < 0.001, unpaired t-test. (**e**) The serum of wild-type and TRIM21-deficient mice infected intraperitoneally with type II *Toxoplasma* was collected at 3 and 7 days post-infection. Cytokine levels were measured using multiplex. Mean ± SEM, *p < 0.05, unpaired t-test.

We previously found TRAF6 and TRIM21 are E3 ubiquitin ligases responsible for the deposition of ubiquitin around inclusions of *Chlamydia trachomatis* and for TRAF6 also around the PV of type II *Toxoplasma* ^22^. While TRAF6 is crucially important for ubiquitin deposition at the PV leading to parasite restriction via p62-dependent GBP-mediated control in infected cells^22^, it is still unclear what the role of TRIM21 is in this process. Additionally, whether these E3 ligases present an important component of the host defence to *Toxoplasma in vivo* has not been investigated. We thus determined the resistance of TRIM21 knockout mice to infection with type II Pru *Toxoplasma*. TRIM21-deficient mice inoculated intraperitoneally with 5 × 10^4^ type II Pru *Toxoplasma* were highly susceptible to infection. At 10 days post-infection, 77% of the TRIM21 knockout mice had succumbed to infection while 83% of the control mice had survived (Fig. 1a). TRIM21 knockout mice exhibited a higher clinical score (characterised by piloerection, hunching and reduced motility) from day 7 post-infection onwards (Fig. 1b). The parasite load assessed by *in vivo* imaging revealed the high susceptibility of TRIM21-deficient mice to *Toxoplasma* acute infection is accompanied with a higher parasite burden 5 days post-infection (Fig. 1c). Additionally, TRIM21-deficient mice that presented poor physical condition typical of infection with *Toxoplasma* exhibited higher tachyzoite parasite counts in their brains compared to healthy wild-type animals on day 7 post-infection (Fig. 1d). Thus, TRIM21 knockout mice are unable to control parasite migration to the brain and/or its replication at this site.

Via interaction and ubiquitination of IRF8, TRIM21 mediates the upregulation of IL-12p40 expression, a cytokine important for innate immunity in macrophages and involved in the stimulation of IFNγ production^39^. TRIM21 has also been shown to downregulate NF-κB signalling in embryonic fibroblasts, leading to lower expression of proinflammatory cytokines^40^. Therefore, we measured the expression of cytokines and chemokines in the serum at day 3 and 7 post-*Toxoplasma* infection. At 3 days post-infection corresponding to the time prior to onset of overt disease, no difference was observed in the levels of serum proinflammatory cytokines. However, at day 7 p.i., corresponding to the time when mice start to succumb to the parasitic infection, TRIM21-deficient mice exhibited significantly decreased levels of RANTES and TNFα (Fig. 1e); both are induced by the NF-κB-mediated signalling pathway. The expression levels of the cytokines investigated were all similar in naïve WT and TRIM21 knockout mice (Supplementary Fig. S1). This suggests TRIM21 mediates resistance to *Toxoplasma* infection *in vivo* by controlling parasite replication and the overt production of some, but not all inflammatory cytokines.

### The E3 ubiquitin ligase TRIM21 localises to GBP1-positive type II Pru vacuoles

**Figure 2:**
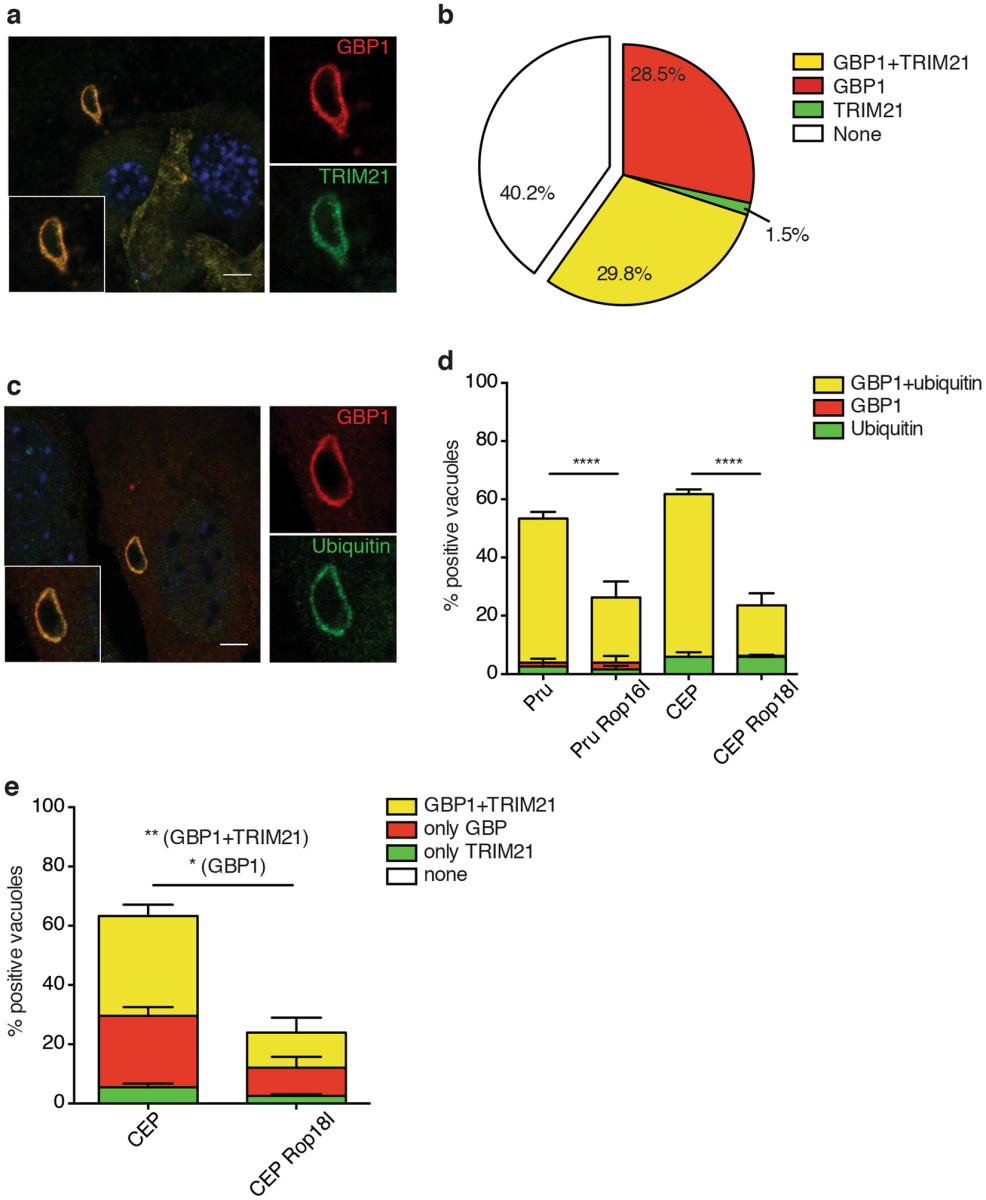
TRIM21 and ubiquitin are targeted to GBP1-positive type II *Toxoplasma* vacuoles in IFNγ-stimulated cells dependent on parasitic virulence factors. (**a**) Representative immunofluorescence confocal images of IFNγ-stimulated mouse embryonic fibroblasts infected 1 h with type II *Toxoplasma* and co-stained for GBP1 and TRIM21. Scale bar is 5 μm. (**b**) Quantification of GBP1 and TRIM21 co-recruitment to the parasite-containing vacuoles 1 h p.i. Data pooled from three independent experiments. (**c**) Representative immunofluorescence confocal images of IFNγ-stimulated mouse embryonic fibroblasts infected 1 h with type II *Toxoplasma* and co-stained for GBP1 and ubiquitin. Scale bar is 5 μm. (**d**) Quantification of ubiquitin- and/or GBP1-positive parasite-containing vacuoles in IFNγ-stimulated mouse embryonic fibroblasts infected 1 h with the canonical *Toxoplasma* type II (Pru) and type III (CEP) strains, as well as with *Toxoplasma* type II strain transgenic for the type I Rop16 (Pru Rop16I), and Toxoplasma type III strain transgenic for the type I version of Rop18 (CEP Rop18I). Data pooled from three independent experiments. Mean ± SEM, ****p < 0.0001, 2-way ANOVA. (**e**) Quantification of TRIM21- and/or GBP1-positive parasite-containing vacuoles in IFNγ-stimulated mouse embryonic fibroblasts infected 1 h with the canonical *Toxoplasma* type III (CEP) strain and *Toxoplasma* type III strain transgenic for the type I version of Rop18 (CEP Rop18I). Data pooled from three independent experiments. Mean ± SEM, *p < 0.05, **p < 0.005, 2-way ANOVA.

Due to the dramatic susceptibility of TRIM21 knockout mice to *Toxoplasma* infection, we investigated the subcellular localisation of TRIM21 in *Toxoplasma*-infected cells. Localisation studies revealed TRIM21 is predominantly found at the type II *Toxoplasma* PV, with up to 26% of vacuoles decorated with the E3 ubiquitin ligase (Supplementary Figs S2a and S3a). As we and others have previously demonstrated, type II *Toxoplasma* is targeted by IRGs^15, 41, 42^, GBPs^43, 44^ (Supplementary Figs S2b and S3b) and ubiquitin^22, 23^ (Supplementary Figs S3c and S4) in an IFNγ-dependent manner, constituting a host defence system that enables the destruction of the PV and the subsequent elimination of the parasite. Using GBP1 as a marker for the host defence system, we showed that TRIM21 and GBP1 are recruited to the same type II *Toxoplasma* PVs in IFNγ-stimulated mouse embryonic fibroblasts (MEFs) (Fig. 2a, Supplementary Fig. S5). Quantitative analysis of the PVs found 29.8% positive for both GBP1 and TRIM21, 28.5% PVs were single-positive for GBP1, while only 1.5% PVs were TRIM21 single-positive (Fig. 2b). Interestingly, TRIM21 was only recruited to half of the PVs that are marked by GBP1, but almost always was recruited to GBP1-positive PVs. These results suggest TRIM21 is preferentially recruited to GBP1-positive type II *Toxoplasma* parasitophorous vacuoles, the same PVs recognised by the host and destined for destruction.

### Parasitic virulence factors prevent recruitment of ubiquitin and TRIM21 to type II GBP1-positive *Toxoplasma* vacuoles

Ubiquitin is a small ubiquitously expressed protein that is a central cytosolic host survival molecule in its ability to tag proteins for either degradation^45^ or to induce immune signalling^46^. In previous work, we showed that ubiquitin targets type II *Toxoplasma* in an IFNγ-dependent manner (Supplementary Figs S3c and S4), leading to TRAF6- and p62-dependent recognition of the PV by GBPs and both factors subsequently mediating parasite clearance^22^. Parasitic virulence factors interfere with this process^22, 43^. As the total percentage of IFNγ-dependent accumulation of ubiquitin around type II *Toxoplasma* PVs was strikingly reminiscent of the recruitment percentage we reported for GBP1^43^, we assessed the colocalisation of the proteins during *Toxoplasma* infection. Both GBP1 and ubiquitin were recruited around the same type II parasites (Fig. 2c). Quantification of GBP1 and ubiquitin recruitment events to type II *Toxoplasma* vacuoles in IFNγ-stimulated mouse embryonic fibroblasts demonstrated that GBP1-positive vacuoles were always positive for ubiquitin. 49% of type II Pru *Toxoplasma* and 56% of type III CEP *Toxoplasma* were decorated with both GBP1 and ubiquitin (Fig. 2d). A minor portion of less than 6% of the PVs was positive for ubiquitin alone, while less than 1% PVs received just GBP1 (Fig. 2d). TRIM21 does not localise to type I *Toxoplasma* (Supplementary Fig. S2a). We previously showed that the recruitment of GBP1, ubiquitin and TRAF6 to type II *Toxoplasma* vacuoles can separately be counteracted by type I *Toxoplasma* ROP16 and ROP18 virulence factors secreted into the host cell cytoplasm^22, 43^. ROP18 has been reported to phosphorylate and hence inactivate the GTPases Irga6 and Irgb6, thereby preventing their recognition of the vacuole^47, 48^. ROP18 can mediate the evasion of GBP1 recruitment to vacuoles of type III parasites. ROP16 localises to the host nucleus and activates STAT3 and STAT6, yet also significantly reduces the recruitment of GBP1 to PVs^43, 49, 50^. In order to determine whether these virulence factors influence the accumulation of the co-recruited GBP1 and ubiquitin in the vicinity of type II *Toxoplasma*, we used the transgenic type II Pru *Toxoplasma* strain expressing the type I version of ROP16 (Pru ROP16_I_), as well as the transgenic type III CEP *Toxoplasma* strain expressing the type I version of ROP18 (CEP ROP18_I_). The transgenic strains Pru ROP16_I_ and CEP ROP18_I_ showed significantly reduced accumulation of the co-recruited GBP1 and ubiquitin compared to their respective parental strains, dropping to 22% and 17%, respectively (Fig. 2d). The less than 6% of PVs that were positive for only ubiquitin were not impacted by the virulence factors (Fig. 2d). These results suggest that *Toxoplasma* virulence factors interfere with the IFNγ-dependent joint recruitment of both GBP1 and ubiquitin to PVs, thus preventing this joint defence machinery from being recruited.

Since GBP1 and ubiquitin colocalisation to type II *Toxoplasma* vacuoles is impaired by the secretion of virulence factors, we investigated whether parasite virulence factors can also alter the recruitment of TRIM21 and GBP1. Immunofluorescence staining for GBP1 and TRIM21 in IFNγ-stimulated MEFs showed both proteins also colocalised to type III CEP *Toxoplasma* strain (Fig. 2e). The transgenic strain CEP ROP18_I_ exhibited significantly reduced accumulation of colocalised GBP1 and TRIM21 compared to its parental strain, decreasing from 34% to 18%, as well as single-positive GBP1 PVs, decreasing from 24% to 10% (Fig. 2e). Interestingly, the proportion of *Toxoplasma* vacuoles decorated with both TRIM21 and GBP1 compared to the proportion of GBP1 single-positive type II vacuoles is approximately conserved (Fig. 2e). This suggests that *Toxoplasma* virulence factors interfere with the recruitment of GBP1 and the colocalised TRIM21 to the parasitophorous vacuoles.

### TRIM21 mediates Lys63-linked ubiquitination recruitment around type II *Toxoplasma*

**Figure 3:**
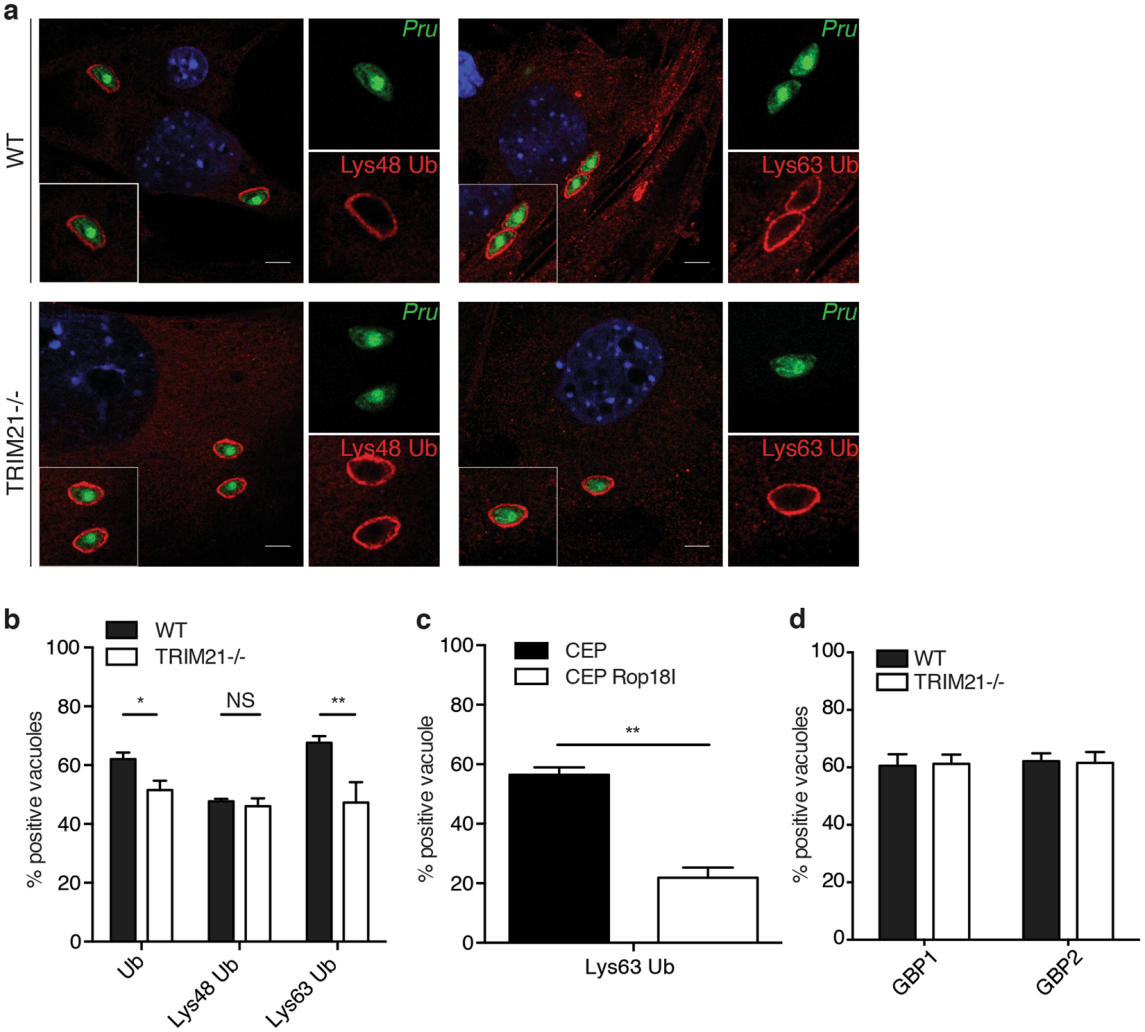
TRIM21 mediates Lys63-linked ubiquitination around type II *Toxoplasma*. (**a**) Representative immunofluorescence confocal images of IFNγ-stimulated wild-type (upper panel) or TRIM21 knockout (lower panel) mouse embryonic fibroblasts infected 1 h with type II *Toxoplasma* expressing GFP and stained for Lys48 ubiquitin (left panel) or Lys63 ubiquitin (right panel). Scale bar is 5 μm. (**b**) Quantification of ubiquitin- (Ub), Lys48 ubiquitin- and Lys63 ubiquitin-positive parasite-containing vacuoles in IFNγ-stimulated wild-type versus TRIM21 knockout MEFs infected 1 h with type II *Toxoplasma*. Data were pooled from three independent experiments. Mean ± SEM, *p < 0.05, **p < 0.005, 2-way ANOVA. (**c**) Quantification of Lys63 ubiquitin-positive parasite-containing vacuoles in IFNγ-stimulated mouse embryonic fibroblasts infected 1 h with the canonical *Toxoplasma* type III (CEP) strain and *Toxoplasma* type III strain transgenic for the type I version of Rop18 (CEP Rop18I). Mean ± SEM, **p < 0.005, 2-way ANOVA. (**d**) Quantification of GBP1 and GBP2 localisation to the parasite-containing vacuoles in IFNγ-stimulated wild-type versus TRIM21 knockout mouse embryonic fibroblasts 1 h p.i. Data pooled from three independent experiments, unpaired t-test.

TRIM21 and ubiquitin were found to both localise to the parasitophorous vacuole of type II Pru *Toxoplasma* (Supplementary Figs S2a, S3a, S3a and S6). Next, we investigated the ubiquitination of type II *Toxoplasma* vacuoles in TRIM21-deficient cells. Distinct ubiquitin linkages have been associated with specific cellular functions. Lys48-Ub substrates have been shown to be targeted for degradation by the proteasome^45, 51^, while Lys63-Ub substrates have been shown to be involved in DNA repair and activation of immune signalling^46, 52^. Thus, we examined the ubiquitin linkage present at the vicinity of type II *Toxoplasma*. Lys48- and Lys-63-linked ubiquitin were observed around type II *Toxoplasma* in both wild-type and TRIM21-deficient cells (Fig. 3a). Quantitative analysis showed TRIM21-deficiency led to a significant decrease in type II *Toxoplasma* vacuolar ubiquitination, dropping from 62 to 52% in TRIM21 knockout cells, suggesting TRIM21-mediated ubiquitination accounts at least in part for the presence of ubiquitin in the vicinity of the parasite (Fig. 3b). TRIM21-deficiency did not affect the Lys48-linked ubiquitination of type II *Toxoplasma* (Fig. 3b). However, Lys63-linked ubiquitination of type II *Toxoplasma* was decreased by one third in TRIM21 knockout cells compared to wild-type cells as the proportion of Lys63-linked ubiquitination dropped from 68 to 47% (Fig. 3b). When expressing type I ROP18 in type II parasites, K63-linked ubiquitination was diminished by 60% concurring with the previously observed lower targeting of TRIM21 and GBP1 (Figs 2d and 3c).

Since TRIM21 is co-localised to the same PVs as GBP1, we asked whether TRIM21 could influence the level of GBP1 and GBP2 recruitment to these PVs. We found that deletion of TRIM21 had no effect on the recruitment percentage of either GBP1 or GBP2 (Fig. 3d). We had previously defined that deletion of chromosome 3 GBPs had no impact on ubiquitin recognition of the type II *Toxoplasma* PV^22^. Thus, our data suggests that the IFNγ-dependent, TRIM21-mediated ubiquitination around the parasites is specific for Lys63-linked polyubiquitin chains.

### TRIM21 mediates restriction and the secretion of inflammatory cytokines during *Toxoplasma* infection

**Figure 4:**
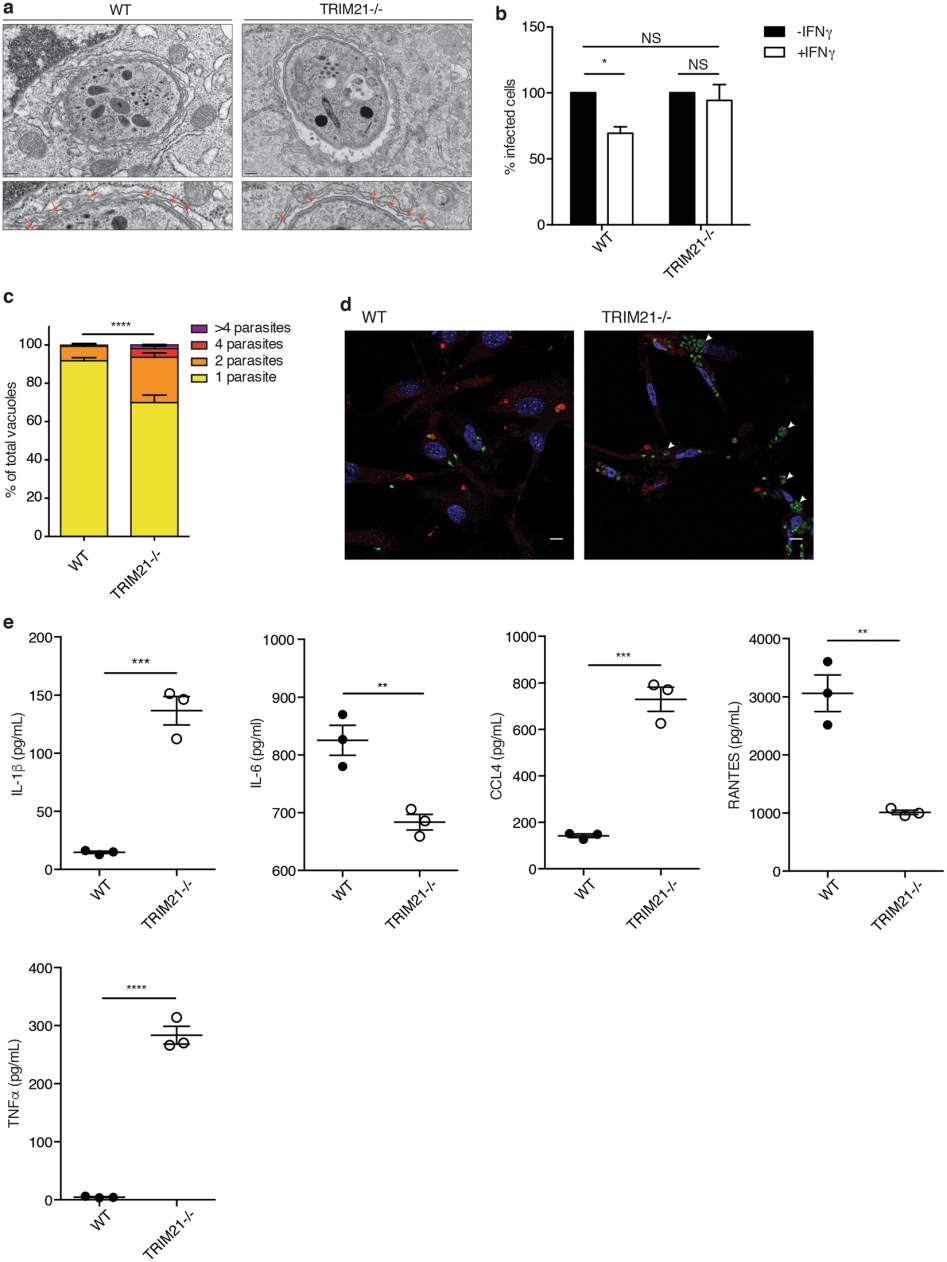
TRIM21 restricts early parasite replication and modulates cytokine production during *Toxoplasma* infection. (**a**) Ultrastructural analysis of type II *Toxoplasma* PVM in IFNγ-stimulated wild-type versus TRIM21 knockout mouse embryonic fibroblasts. The lower images are enlarged views from the upper images. Red arrows indicate blebbing and disruption of the parasitophorous vacuole. Scale bar is 200 nm. (**b**) Percentage of infection with *Toxoplasma* in untreated or IFNγ-stimulated TRIM21 knockout and wild-type cells at 1 h p.i.. Data pooled from three independent experiments. Mean ± SEM, p < 0.05, 2-way ANOVA. (**c**) Quantification of the number of parasites per vacuole in IFNγ-stimulated MEFs infected for 15 h with type II *Toxoplasma*. Invaded *Toxoplasma* were assessed for GBP1 recruitment. Data pooled from three independent experiments. Mean ± SEM, ****p < 0.0001, 2-way ANOVA. (**d**) Representative confocal images of replicating type II *Toxoplasma* in IFNγ-stimulated MEFs at 15 h post-infection. GBP1 staining in red, type II *Toxoplasma* in green. White arrows indicate highly replicative *Toxoplasma* vacuoles. Scale bar is 10 μm. (**e**) Cytokine production in culture supernatants of IFNγ-stimulated wild-type versus TRIM21-deficient MEFs infected with type II *Toxoplasma* for 18 h. Data representative of three experiments. Mean ± SEM, **p < 0.01, ***p < 0.005, unpaired t-test.

We next investigated the downstream effects of TRIM21-mediated type II *Toxoplasma* ubiquitination. Mallery *et al*. showed that TRIM21 binds to invading antibody-coated adenoviruses and targets them to degradation by the proteasome due to its E3 ligase activity^34^. More recently, it has been shown that TRIM21 also restricts *Salmonella* intracellular infection^36^. We thus assessed whether TRIM21-mediated ubiquitination of type II *Toxoplasma* enables restriction of intracellular parasitic infection. Cell-autonomous restriction of *Toxoplasma* in IFNγ-stimulated cells is characterised by the blebbing and disruption of the PV^15, 16, 53^. Some GBPs, including GBP1, are required for this phenomenon^17, 44^. We thus investigated by transmission electron microscopy the integrity of the vacuolar membrane of type II *Toxoplasma* in IFNγ-stimulated wild-type or TRIM21 knockout MEFs 2 h after infection. Disruption and blebbing of the vacuolar membrane of type II *Toxoplasma* was not markedly impaired in TRIM21-deficient cells (Fig. 4a), suggesting TRIM21 is not involved in the IFNγ-induced rupture of *Toxoplasma* PVs. *In vivo*, we had observed an increase in parasite load during the acute phase of infection in multiple sites (Fig. 1c,d). We thus asked whether TRIM21 knockout cells would exhibit an increased relative percentage of infected cells in IFNγ-treated versus untreated cells at 24 h p.i. compared to the wild-type cells. Indeed, only control cells showed significantly less infected cells following IFNγ stimulation (Fig. 4b). As another measure of parasite restriction of *Toxoplasma* replication in TRIM21-deficient cells, enumeration of the parasites per vacuole showed the E3 ubiquitin ligase controled the replication of the parasites (Fig. 4c). At 15 h post-infection, *in vitro* vacuoles containing 2 or more parasites were significantly more abundant in cells lacking TRIM21 (Fig. 4c). Equally, representative microscopy images demonstrated the increased replicative capacity of type II *Toxoplasma* in IFNγ-stimulated TRIM21 knockout versus wild-type MEFs (Fig. 4d). These results indicate TRIM21 is involved in early restriction and later elimination of *Toxoplasma*.

TRIM21 has also been ascribed innate immune signalling function. TRIM21 mediates the upregulation of IL-12p40 expression via interaction with and ubiquitination of IRF8, a cytokine important for innate immunity in macrophages and involved in the stimulation of IFNγ production^39^. Additionally, TRIM21 has also been shown to modulate NF-κB signalling in MEFs, leading to differential expression of proinflammatory cytokines, especially IL-6^35, 40, 54^. We hypothesised that TRIM21 in part confers resistance to *Toxoplasma* infection by controlling cytokine levels to mediate non-cell-autonomous immune responses. Therefore, we measured the expression of cytokines analogous to our *in vivo* study. We determined a decreased secretion of IL-6, chemokine (C-X-C motif) ligand 1 (CXCL1), CXCL3, leukemia inhibitory factor (LIF) and RANTES, as well as an increased production of IL-1α, IL-1β, chemokine (C-C motif) ligand 3 (CCL3), CCL4, CCL11, granulocyte-macrophage colony-stimulating factor (GM-CSF) and TNFα in INFγ-stimulated TRIM21-deficient cells after infection with type II *Toxoplasma* (Fig. 4e, Supplementary Fig. S7). The differential cytokine expression observed here could either be solely due to the TRIM21 deficient genotype, but could also be impacted by IFNγ stimulation and/or *Toxoplasma* infection alone. However, TRIM21 alone (PBS-only) has been shown not to intrinsically have an impact on the production of inflammatory cytokines^35^ and we observed no difference in cytokine expression in naïve mice (Supplementary Fig. S1). These results demonstrate that TRIM21 contributes to the restriction of *Toxoplasma* infection, also acting either directly or indirectly as an innate immune modulator that regulates the inflammatory cytokine responses *in vitro*.

## Discussion

Our study has identified TRIM21 as a novel innate immune effector for the eukaryotic pathogen *Toxoplasma gondii*. To date, only the inflammasome and TLR11/12 are known to recognise *Toxoplasma*, signal to the immune system and protect against the infection *in vivo*. TRIM21 expands this concept to E3 ubiquitin ligases and opens the door to studying how distinct ubiquitin-driven cascades signal to activate innate immune defence systems. The balance between innate immune-driven detection and pathogen elimination on one hand and *Toxoplasma* replication and progression of the infection to chronicity on the other are central to the successful propagation of the parasite.

E3 ubiquitin ligases have emerged as important mediators of resistance against intracellular bacteria by regulating cell-autonomous restriction. LRSAM1 directly recognises *Salmonella*, leading to ubiquitination of the bacterium and recruitment of LC3-positive autophagosomes via the autophagy receptor NDP52^26^. TRAF6 partially mediates the decoration of *Chlamydia* inclusions with ubiquitin, thereby regulating GBP recruitment to the vacuoles for successful bacterial clearance^22^. TRAF6 is also central for the cell-autonomous restriction of *Toxoplasma* ^22^. Activation of this *Toxoplasma*-restricting E3 ubiquitin ligase is necessary for the effective production of proinflammatory cytokines during the parasitic infection *in vitro* ^31^. Regardless, neither the effect of LRSAM1 nor TRAF6 have been investigated during a bacterial or protozoan infection *in vivo*. *In vivo*, Parkin knockout mice are highly susceptible to *Mycobacterium tuberculosis* infection exhibiting a higher bacterial load and HOIL-1 knockout mice succumb to *Citrobacter rodentium, Listeria monocytogenes* and *Toxoplasma* infection, exhibiting unchecked parasite and *Listeria* burden^28^. Additionally, HOIL-1 knockout mice present an impaired production of protective cytokines by macrophages during *Listeria* infection, but it is unclear whether this E3 ubiquitin ligase also regulates the innate response to *Toxoplasma* and whether this E3 ligase directly recognises the parasite^28^. TRIM21 restricts *Salmonella typhimurium* growth and targets adenoviruses to degradation by the proteasome^34–36^ and *in vitro* mediates the formation of Lys63-linked chains to upregulate IRF3, IRF5, IRF7, NF-κB and AP-1, thereby inducing the production of proinflammatory cytokines^35^. *In vivo*, TRIM21 knockout mice are susceptible to adenoviral challenge, a property dependent on the capability of TRIM21 to restrict viral replication and to induce a cytokine response^37, 38^. Whether TRIM21 restricts bacterial infections *in vivo* remains to be investigated.

Here, we show that TRIM21 localises to the type II *Toxoplasma* vacuole and is required for the production of cytokines as well as the parasite restriction during *Toxoplasma* infection. The modulation of cytokine production following TRIM21 deficiency appears to differ at the cellular level compared to *in vivo*. This can be explained by both the timing of infection (3–7 days *in vivo* and 24 h *in vitro*) as well as the cell types involved in the production of the cytokines in both environments. It is of note that *in vitro* IFNγ stimulation and/or *Toxoplasma* infection could impact cytokine production. Importantly, *in vivo* as seen in naïve mice, TRIM21 deficiency alone does not impact the expression of inflammatory cytokines.

The E3 ubiquitin ligase HOIL-1 is responsible for lowering *Toxoplasma* burden during *in vivo* infection, but the intracellular localisation of HOIL-1 during the infection remains unknown^28^. The role of TRIM21 has only been studied *in vivo* during viral infections^37, 38^. The action of E3 ubiquitin ligases during parasitic infection remain unclear. Our studies places TRIM21 as an E3 ubiquitin ligase that mediates both clearance of a eukaryotic pathogen and the successful immune response required for *in vivo* host survival. The IFNγ-dependent restriction of *Toxoplasma* replication could be mediated directly by TRIM21, or the E3 ligase could induce other interferon-stimulated genes (such as other members of the TRIM family) to regulate parasitic clearance. As some atypical strains of *Toxoplasma* ^55^ and TRIM21^56, 57^ have been shown to modulate the secretion of type I interferons, which can in turn stimulate the production of TRIMs, it would be of interest to study the production of type I IFNs in TRIM21 deficient cells during the course of *Toxoplasma* infection. Equally, *Toxoplasma*-infected TRIM21 deficient cells or mice might present with increased levels of other TRIM family member proteins as demonstrated for poly I:C and LPS-stimulated fibroblasts^40^.

TRIM21 is one of the molecules operating with GBP1 at the type II and III *Toxoplasma* vacuole. As with the recruitment of GBP1, the localisation of TRIM21 to the PV can be diminished by the expression of type I Rop16 and Rop18^43^. As Rop18 has been found at the *Toxoplasma* PV where it phosphorylates immunity-related GTPases preventing their recruitment^58, 59^, it is highly likely that TRIM21 is another player in this cohort of host defence molecules^22^. Rop16 localises to the host nucleus activating STAT3/6, thus its direct effect on TRIM21 localisation is less clear. It is still conceivable that Rop16 may phosphorylate TRIM21 or an as yet unknown host substrate, which then hinders recruitment of the E3 ubiquitin ligase to the PV. Regardless, both type II and III parasites recruit IRGs and GBPs to their vacuoles, and Rop16 is expressed by type I and III *Toxoplasma*. Thus it is likely that STAT3/6 activation by Rop16 of type I and III *Toxoplasma* and consequential inhibition of pro-inflammatory cytokine secretion or other STAT3/6-mediated effects decreases the virulence and supersedes interference with the IRG-mediated cell-autonomous immunity.

TRIM21 it is undoubtedly not the sole E3 ubiquitin ligase depositing ubiquitin at the vicinity of the parasite, as can be deduced from the remaining ubiquitination level in TRIM21-deficient cells. Interestingly, TRIM21 deletion does not impact the percentage of PVs recognised by GBP1 and GBP2, however, it does have an impact on Lys63-linked ubiquitination in its vicinity. We have recently identified TRAF6 as another E3 ubiquitin ligases at the PV^22^. Importantly, TRIM21 and TRAF6 in combination certainly also do not comprise the full set of E3s at the PV and it will be imperative to identify additional ligases acting directly in the vicinity of the parasite. Further studies need to characterise and understand the recruitment kinetics and targets of the ubiquitin ligases at *Toxoplasma* vacuoles.

Following infection with adenovirus and *Salmonella*, TRIM21 catalyses the formation of K48 and K63 ubiquitin chains^34, 35^. The presence of K48 ubiquitin chains targets the virions for degradation by the proteasome in dependence of the AAA ATPase VCP^34, 60^. The synthesis of K63 ubiquitin chains triggers innate immunity signalling via activation of the NF-κB, IRF3/5/7 and AP-1 pathways, leading to an antiviral state characterised by the production of inflammatory cytokines^35^. In contrast to bacterial and viral infections, we show that TRIM21 impacts the formation of only K63 and not K48 ubiquitin chains at the vicinity of *Toxoplasma* PVs, concomitant with the activation of the innate immune signalling characterised by the production of inflammatory cytokines. TRIM21 comprises three N-terminal protein domains: a RING domain involved in protein-protein interactions that confers it with an E3 ligase activity, two B-box domains that are zinc-binding motifs and a helical coiled-coil domain important for protein-protein interactions^61^. It is tempting to speculate that the E3 ligase activity of TRIM21 directly mediates the ubiquitination at the vicinity of *Toxoplasma*. TRIM21 ubiquitination substrates are currently unknown and it will be important to define such substrates to assess whether the E3 ubiquitin ligase asserts its effect through host proteins or parasite proteins.

TRIM21 is additionally characterised by a C-terminal B30.2/PRYSPRY domain that was shown to strongly bind to the Fc part of antibodies^54, 62, 63^. Mallery *et al*. have disputed the dogma that antibodies do not have an intracellular function by reporting that TRIM21-mediated neutralisation of adenovirus is dependent on the presence of intracellular antibodies coating the invading particles^34^. *In vivo*, As far as *Toxoplasma* is concerned, the presence of antibodies inside the vacuole at the surface of the parasite following invasion remains unclear. It is generally accepted that a fraction of the *Toxoplasma*-derived surface-exposed proteins are shed or not carried into the cell due to sieving at the Moving Junction during parasite invasion and interestingly antibody-binding of surface AMA1 blocks parasite invasion^64, 65^. Nevertheless, immunoelectron microscopy of anti-P30 (the major *Toxoplasma* surface protein) coated parasites left to invade HeLa cells demonstrated that while most of the parasite surface-attached antibody was shed, some escape was noted and a minor fraction of the parasites were faintly labelled with anti-P30 antibody intracellularly^66^. In terms of viral neutralisation, it has been calculated that 1.6 antibodies per virus particle suffice for TRIM21 activity in the presence of interferon^67^. It is thus conceivable that very few residual antibodies bound to the parasite could trigger the activity of TRIM21. However, neither the molecular mechanism of antibody-triggered TRIM21 activity has been elucidated, nor do we know how much residual antibody of which specificity the parasite is able to carry into invaded cells *in vivo*. Recruitment of TRIM21 to the PV is most likely independent of antibody binding as the PV is disrupted at two hours post-infection, while we can localise TRIM21 at the PV already after one hour^53^. Thus, even though difficult to realise practically, it remains to be investigated if the subsequent effects exerted by TRIM21 during a *Toxoplasma* infection *in vivo* are dependent on intracellular antibody-binding.

The requirement of functional intracellular recognition systems -such as the Toll-like receptors (TLRs), inflammasome and autophagy- during the *in vivo* acute phase of *Toxoplasma* infection is well-established. Mice lacking TLR12, the TLR-induced adaptor proteins MyD88 or UNC93B1 and the inflammasome proteins NLRP3 or ASC all exhibit a high susceptibility to *Toxoplasma* infection during the early stage of the infection due to an impaired innate immune response^68–71^. Moreover, mice deficient in the autophagy proteins ATG5 or ATG16L1 as well as in the E3 ubiquitin ligase HOIL-1 fail to restrict *Toxoplasma* replication and succumb to acute infection^28, 72, 73^. Our findings show that TRIM21-deficient mice exhibit death at 7 days post-infection, placing TRIM21 into the category of these major intracellular acute phase mediators of *Toxoplasma* resistance. Contrary to the E3 ligase HOIL-1, TRIM21 mediates both immune activation and parasite restriction during an *in vivo Toxoplasma* infection^28^. We observe a higher parasite burden in the brain of TRIM21 knockout mice, indicating this E3 ligase is also crucial for *Toxoplasma* restriction. However, our results indicate TRIM21 also plays a role in the induction of proinflammatory cytokines concurrent with protection of infected mice. Thus, E3 ubiquitin ligases are a new category of acute phase resistance mediators during *Toxoplasma* infection, capable of both parasite restriction and innate immune induction. The ultimate death of TRIM21 knockout mice could be partly mediated by the portion of TRIM21 localised at the PV, but also by an unidentified pool of TRIM21 in the cytoplasm. We speculate that the TRIM21-induced inflammatory response observed both *in vitro* and *in vivo* is an important role of TRIM21 during *Toxoplasma* infection.

In summary, we have identified TRIM21 as a novel intracellular innate immune mediator for the eukaryotic pathogen *Toxoplasma*. This defines ubiquitination as a key process of innate immune recognition pathways for *Toxoplasma*. It additionally presents the vital E3 ubiquitin ligase driving not only parasite restriction, but also innate immune activation while localising to the vacuole of an intracellular protozoan. TRIM21 is thus a new key effector molecule in the IFNγ-induced resistance to *Toxoplasma* acting in concert with the GBP-mediated defence machinery. This complex mechanism between TRIM21 and GBP1 highlights a new host defence mechanism against this intracellular pathogen, where TRIM21 stimulates innate immune defence during *Toxoplasma* infection. The specific molecular mechanisms underlying the newly discovered *Toxoplasma* resistance factor TRIM21 *in vivo* remains to be deciphered.

## Methods

### Ethics statement

All procedures involving mice were approved by the local ethical committee of the Francis Crick Institute Ltd, Mill Hill Laboratory and are part of a project license approved by the Home Office, UK, under the Animals (Scientific Procedures) Act 1986. All experiments were performed in accordance with relevant guidelines and regulations.

### Mice and infection

Wild-type (WT) and TRIM21 deficient^40^ mice on the C57BL/6 background were bred and intercrossed at the Francis Crick Institute Ltd, Mill Hill Laboratory under specific pathogen-free conditions. To minimise genetic divergence between transgenic mice and their wild-type controls, WT mice were derived from the TRIM21 heterozygous. Experiments were performed on 6- to 8-week-old females. For infections, *Toxoplasma* type II strain Pru expressing GFP and firefly luciferase^6, 43, 74^ were harvested from the peritoneum of a 4-day infected C57BL/6 mouse and passaged through a 26 gauge needle (BD). Parasites were washed twice with PBS and mice were injected intraperitoneally (i.p.) with 5 × 10^4^ parasites suspended in PBS. Since TRIM21 has been shown to bind to Fc regions of antibodies, this was to make sure our *in vivo* infections were performed with *Toxoplasma* tachyzoites that had a natural coat of antibodies on them. For *in vivo* imaging, mice were injected i.p. with 3 mg firefly D-luciferin (Perkin Elmer), left for 10 min and imaged with an IVIS Spectrum-bioluminescent and fluorescent imaging system (Xenogen Corporation) under isoflurane anaesthesia (Abbott).

### Cell culture and infection

Human foreskin fibroblasts (HFFs, ATCC), Raw 264.7 macrophages (ATCC) and primary mouse embryonic fibroblasts (MEFs, home-made) were cultivated in Dulbecco’s Modified Eagle Medium (DMEM, Gibco) supplemented with 10% fetal bovine serum (FBS, Gibco) at 37 °C in the presence of 5% CO_2_. Experiments were performed at a multiplicity of infection (MOI) of 1–10 after induction with 100 U/mL murine recombinant IFNγ (R&D Systems) for 16 h.

### Parasite culture

Unless stated otherwise, *Toxoplasma gondii* type I strain RH wild-type or RH expressing tdTomato, and *Toxoplasma* type II strain Prugniaud (Pru) wild-type or Pru expressing GFP and firefly luciferase were used (kind gifts from Marc-Jan Gubbels and Jeroen Saeij)^43, 74^. All strains of *Toxoplasma gondii* were maintained by serial passage on monolayers of HFFs as described previously^75^.

### Antibodies

Primary antibodies used for immunofluorescence were: rabbit polyclonal anti-GBP1^17, 43^, goat polyclonal anti-TRIM21 (#sc-21365, Santa-Cruz Biotechnology), mouse monoclonal anti-ubiquitin FK2 (#PW8810, Enzo Life Sciences), rabbit monoclonal anti-ubiquitin Lys48 (#05-1307, Merck Millipore), rabbit monoclonal anti-ubiquitin Lys63 (#05-1308, Merck Millipore) and anti-GAPDH (#2118 S, Cell Signaling). Secondary antibodies for immunofluorescence were from Molecular Probes: AlexaFluor®488 goat anti-rabbit (#A11034), AlexaFluor®488 donkey anti-goat (#A11055), AlexaFluor®594 goat anti-rabbit (#A11037) and AlexaFluor®594 donkey anti-goat (#A11058).

### Immunofluorescence microscopy

Immunofluorescence microscopy was performed on 12 mm, #1.5 coverslips (Thermo Fisher) in 24-well plates (Nunc). Cells were stimulated with 100 U/mL recombinant murine IFNγ (R&D Systems) overnight and infected with *Toxoplasma* at an MOI of 5–10. After the indicated infection time, cells were washed 3 times with PBS and fixed in 3% paraformaldehyde (Sigma Aldrich) at RT for 20 min. Cells were permeabilised for 15 min at RT with 50 mM NH_4_Cl/0.2% saponin/PBS and kept at 4 °C for up to a week in 0.2% fish skin gelatin/0.02% saponin/0.02% NaN_3_/PBS (PGAS) until antibody staining. Briefly, coverslips were incubated with appropriate primary antibody in a humid chamber at RT for 1 h. Coverslips were then washes 3 times in PGAS and subsequently incubated with appropriate secondary antibody in a humid chamber at RT for 1 h in the dark and washed 3 times in PGAS solution followed by 3 washes in PBS. Nuclei were stained with 1 μg/mL Hoechst 33442 solution (Sigma-Aldrich). Coverslips were mounted on Superfrost® Plus glass slides (Thermo Scientific) with 50 µL Mowiol® 4–88 solution (Sigma-Aldrich) and kept in the dark at 4 °C. The frequency of *Toxoplasma*-positive vacuoles was determined by counting 100 PVs per replicate using an AxioPlan II fluorescent microscope (Carl Zeiss) equipped with DAPI, GFP and rhodamine filters. Images were captured with a Leica TCS-SP5 inverted confocal microscope (Leica Microsystems) fitted with conventional photomultiplier tubes and hybrid detectors and using a 100x Leica HCX PL APO CS (numerical aperture 1.4) oil immersion objective or with Nikon TI-E inverted epifluorescent microscope fitted with an orca flash 4 camera and a SpectraX light source. Images were processed in Fiji/ImageJ software (National Institute of Health).

### Transmission electron microscopy

Ultrastructural analysis of IFNγ-stimulated, *Toxoplasma*-infected wild-type or TRIM21 knockout MEFs infected with type II *Toxoplasma* for 2 h. Cells were washed 3x in PBS before 0.05% trypsin-EDTA treatment. Samples were prepared according to details further described in Supplemental Information. Samples were observed with a JEOL 1200 EX transmission electron microscope (JEOL, Tokyo, Japan) equipped with an Orius 1000 CCD camera (Gatan, Pleasanton, CA, USA).

### Replication assay

Wild-type or TRIM21 knockout MEFs were seeded on 12 mm, #1.5 coverslips in 24-well plates and induced with 100 U/mL IFNγ 16 h before infection. Cells were infected with type II *Toxoplasma* for 18 h at an MOI of 5 and subsequently fixed and permeabilised as described before. The number of parasites per vacuoles was determined by counting 50 to 100 PVs per technical replicate using a Nikon TI-E fluorescent microscope. Images were captured using a 60x Nikon oil immersion objective. Images were processed in Fiji/ImageJ software.

### Relative infection assay

Wild-type or TRIM21 knockout MEFs unstimulated or stimulated with 100 U/mL IFNγ overnight were infected with *Toxoplasma* Pru-tdTomato at an MOI of 3 for 24 h. Cells were washed with PBS, 2 mM EDTA, 1%BSA and harvested for analysis by flow cytometry. Uninfected samples were used as a gating control and infected samples without IFNγ-treatment were used as a normalisation control to determine the relative infection (%) of IFNγ-treated samples.

### Quantitative polymerase chain reaction

Wild-type or TRIM21 knockout MEFs were stimulated with 100 U/mL IFNγ overnight and infected with type II *Toxoplasma* for 1 h. Cellular RNA was extracted using the miRNeasy kit (Qiagen) and reverse transcribed into cDNA using the SuperScript® VILO^TM^ cDNA Synthesis kit (Thermo Fischer Scientific). The quantitative PCR was performed using the TaqMan® Real-Time PCR Assay system (Applied Biosystems) with *Gbp1* (Mm00657086_m1), *Gbp2* (Mm00494576_g1) and *Gapdh* (Mm99999915_g1) primers.

### Quantification of cytokines

For serum cytokines, blood was collected at day 3 and 7 post-infection from the saphenous vein or by terminal cardiac puncture, respectively. Serum was obtained after coagulation at RT for 30 min and centrifugation at 4 °C for 10 min at 1000 g. Serum cytokines and chemokines analysis was performed by mouse 32-plex Discovery Assay® (Eve Technologies, Calgary, Alberta, Canada). For cell supernatant cytokines, wild-type or TRIM21 knockout MEFs were stimulated with 100 U/mL IFNγ overnight and infected with type II *Toxoplasma* for 18–24 h. Supernatants were collected and analysed by enzyme-linked immunosorbent assay (ELISA) or mouse 32-plex Discovery Assay®. Commercially available kits were used according to the manufacturer’s instructions to quantify the concentration of CCL4 (Insight Bioscience), IL-1β (BD Biosciences), IL-6 (eBioscience), RANTES (eBioscience) and TNFα .

### Statistical analysis

All statistical significance analyses were performed using Prism software (GraphPad Software). Comparisons of data were performed with unpaired Student’s *t*-test or using two-way ANOVA with Sidak multiple comparison correction. Survival rates were compared by log-rank survival analysis of Kaplan-Meier curves.

## Electronic supplementary material

Supplementary information
