# Supplementary material for: TRIM21 is critical for survival of Toxoplasma gondii infection and localises to GBP-positive parasite vacuoles
Source: Sci Rep. 2017 Jul 12;7:5209. doi: 10.1038/s41598-017-05487-7 (PMC5507857; doi:10.1038/s41598-017-05487-7)

TRIM21 is critical for survival of *Toxoplasma gondii* infection and localises to GBP-positive *parasite* vacuoles

Clémence Foltz, Anna Napolitano, Rabia Khan, Barbara Clough, Elizabeth M. Hirst and Eva-Maria Frickel*

Supplementary Information

Supplementary Methods

Transmission electron microscopy

After centrifugation at 900g for 5 min, cells were immersion fixed in 2% glutaraldehyde (Agar Scientific)/2% paraformaldehyde in 0.1M sodium cacodylate buffer, pH7.2, embedded in 2% agarose and centrifuged for 3 min at 13000rpm before being returned to 2% glutaraldehyde/2% paraformaldehyde in 0.1M sodium cacodylate buffer, pH7.2 overnight. Samples were washed in sodium cacodylate buffer 0.1M pH7.2 (SCB) for 10 min, post-fixed in 1% osmium tetroxide/SCB for 1.5h, washed in SCB for 10 min and stained on bloc in 1% aqueous uranyl acetate for 1.5h. Samples were dehydrated in 50% ethanol, 75% ethanol and 90% ethanol for 10 min each, 3x 10 min in 100% ethanol and 2x 30 min in propylene oxide. Samples were embedded in epon resin (Agar Scientific, Stansted, UK) for 5 changes over 8h and polymerised at 70°C overnight. 50nm sections were mounted on pioloform-coated slot grids and stained with uranyl acetate for 30 min and with Reynold's lead citrate for 7 min.

Supplementary Figure 1

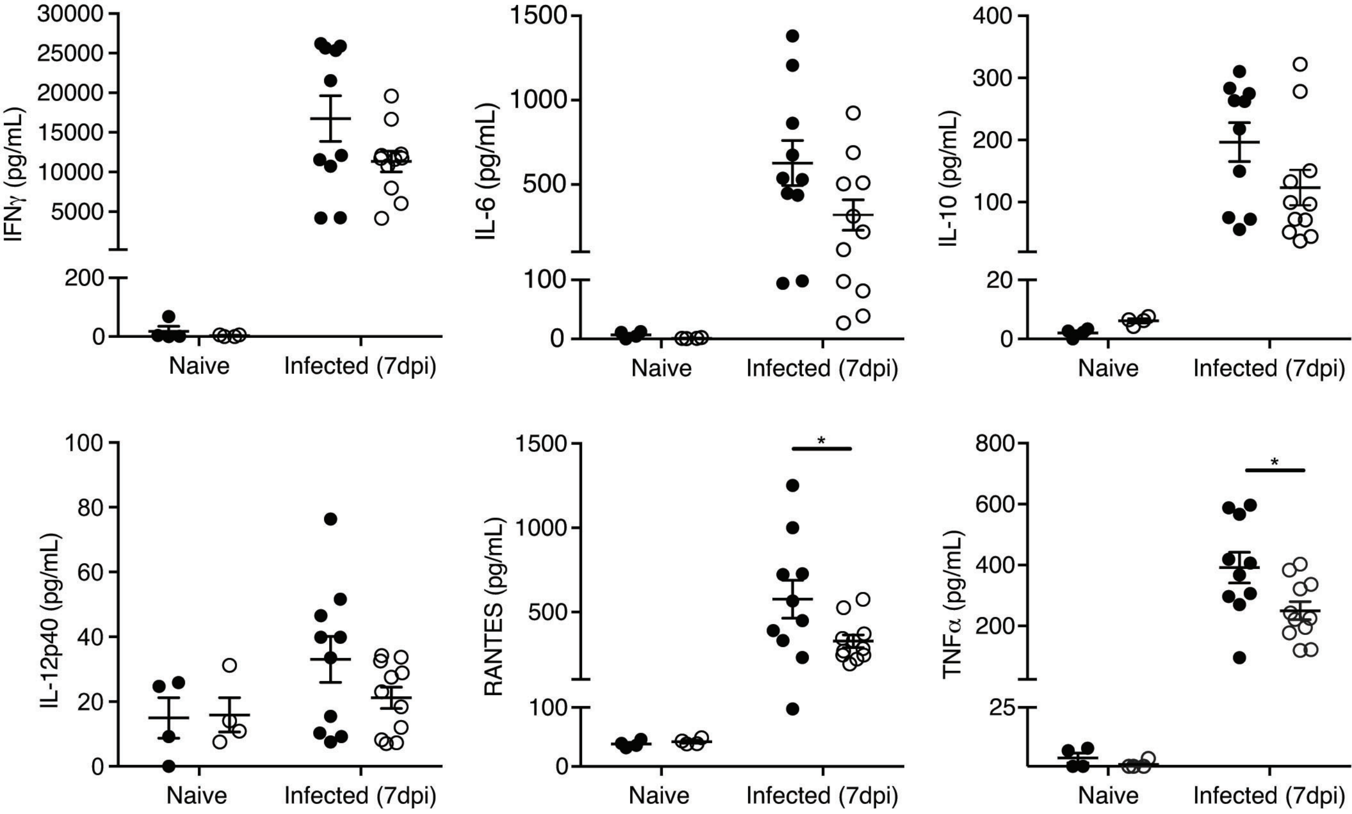

Supplementary Figure 2

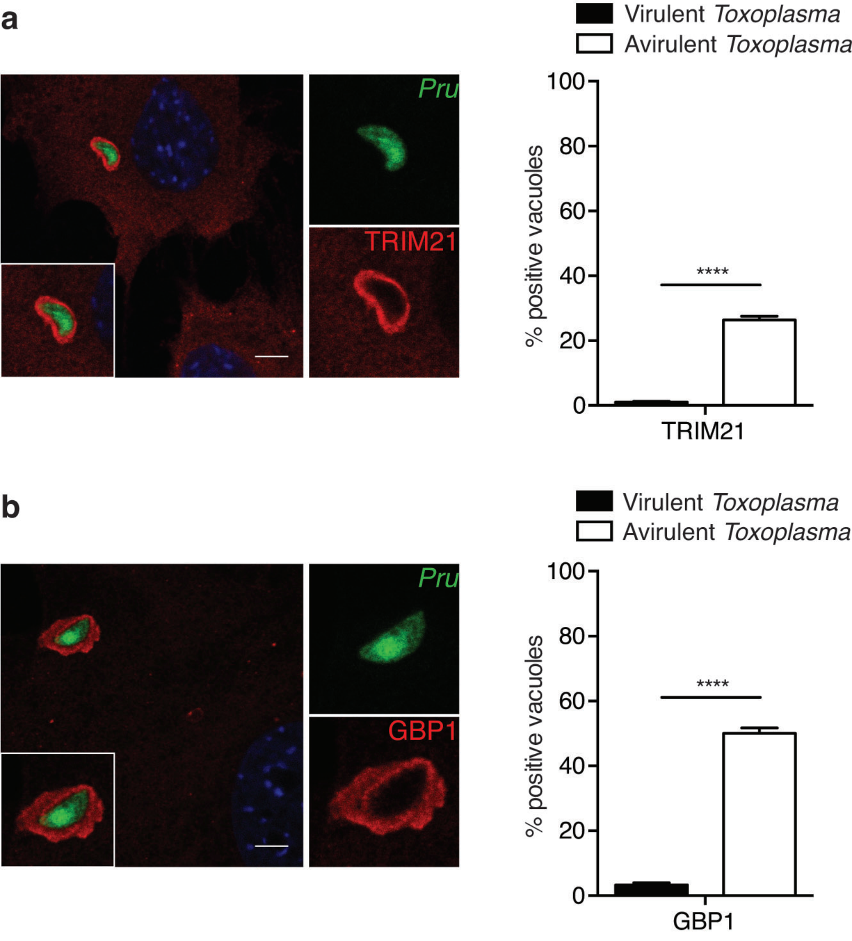

Supplementary Figure 3

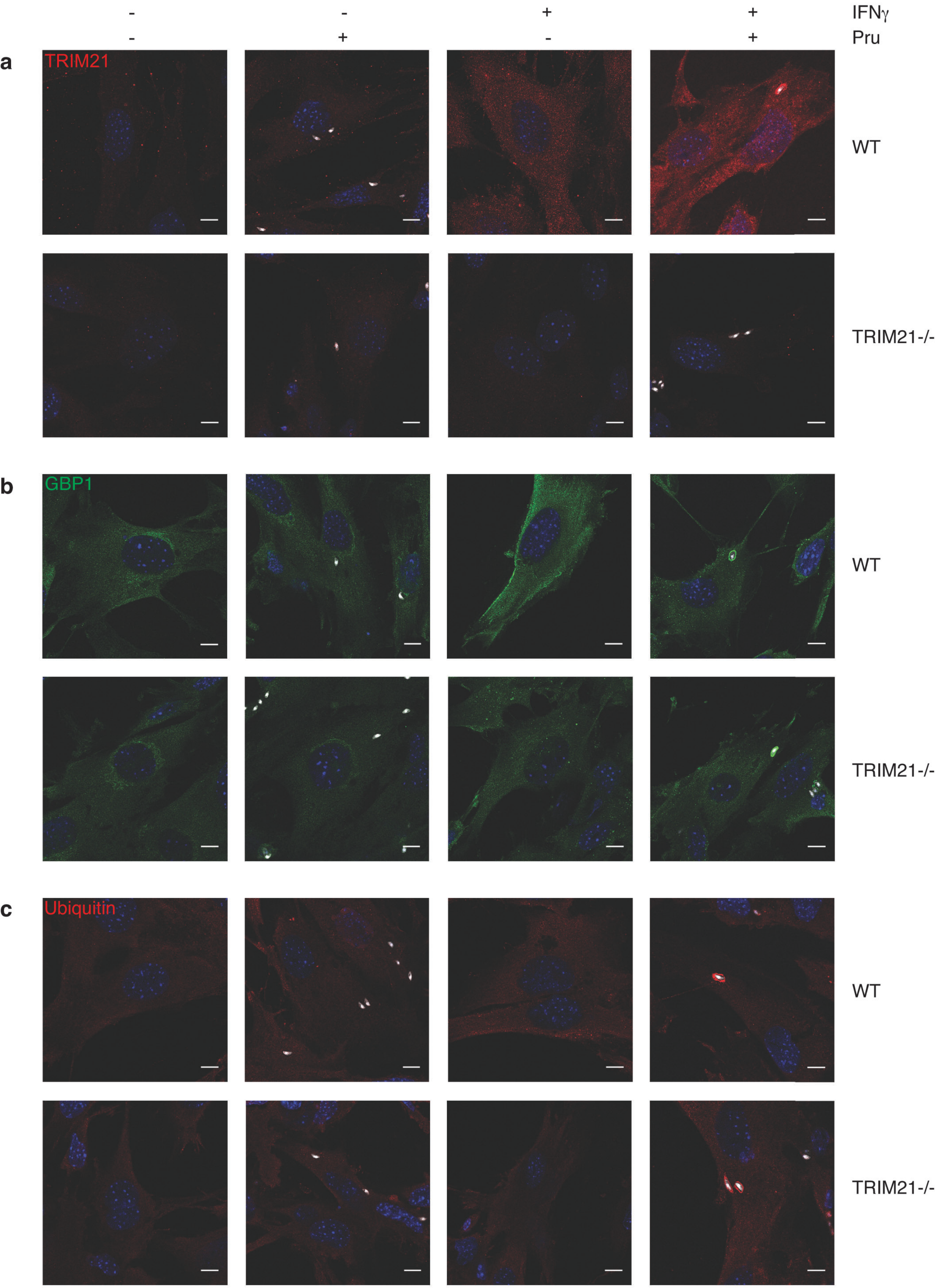

Supplementary Figure 4

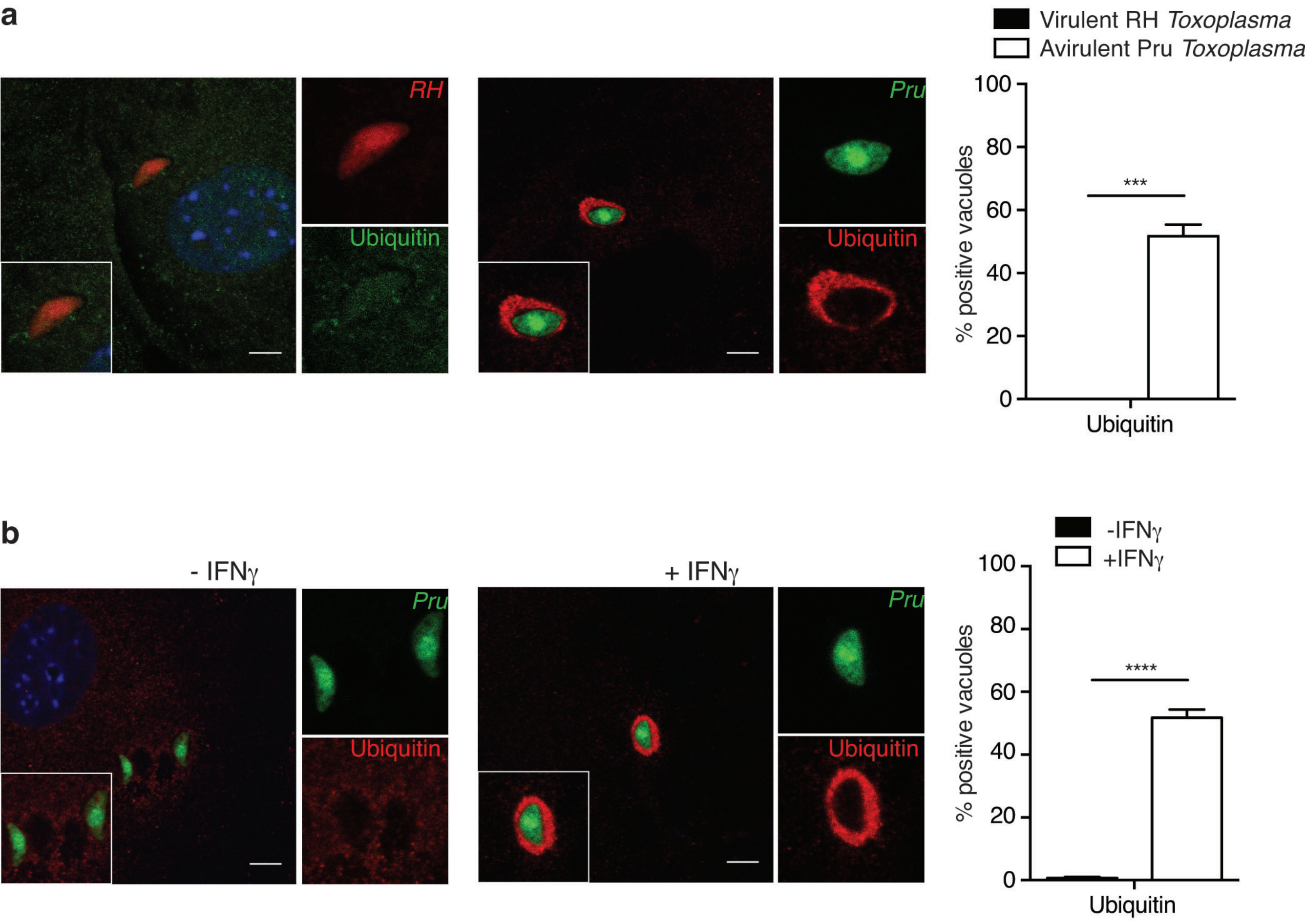

Supplementary Figure 5

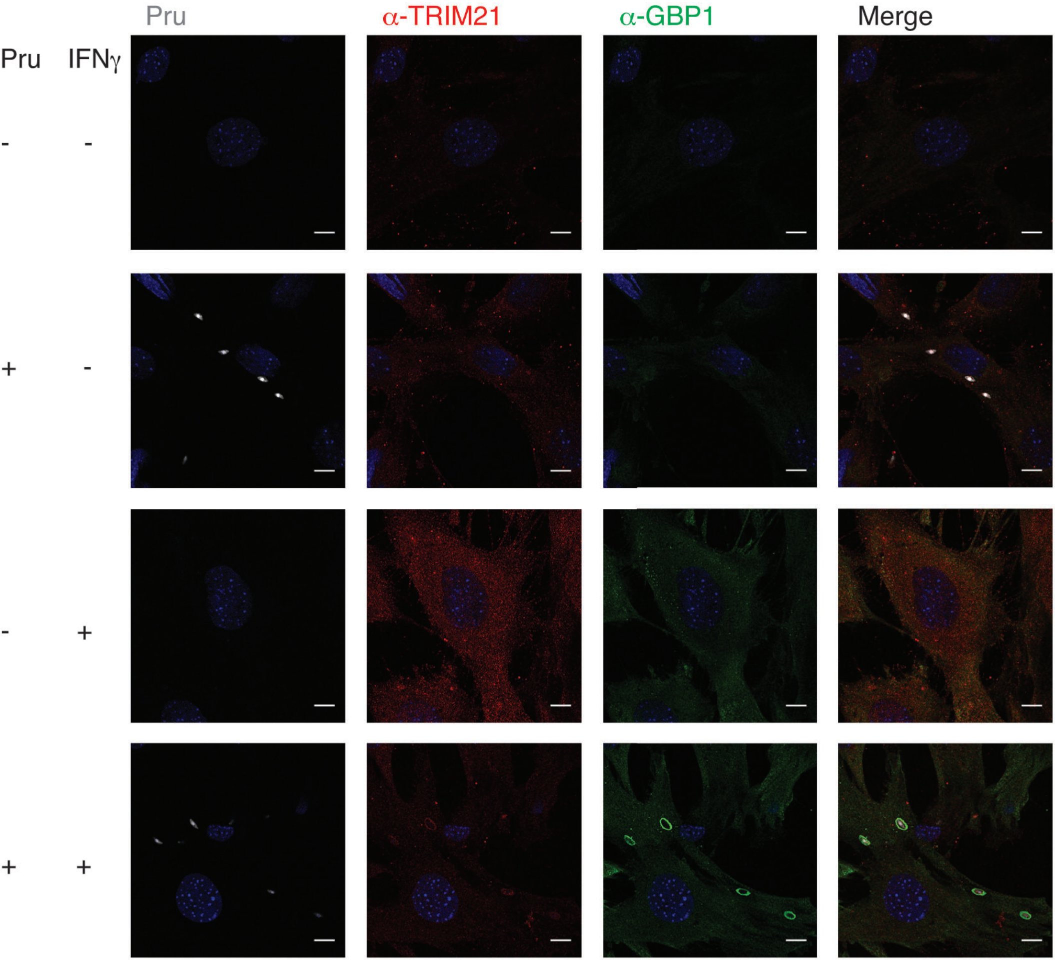

Supplementary Figure 6

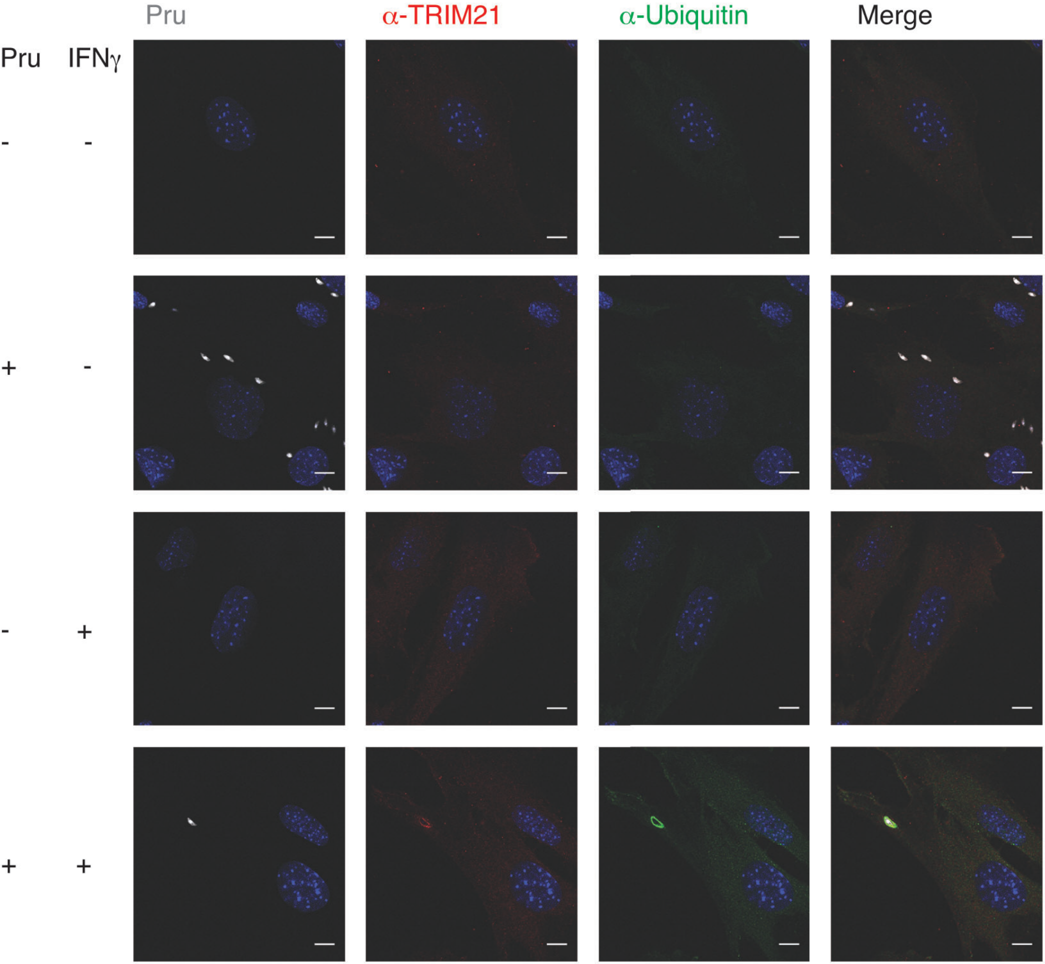

Supplementary Figure 7

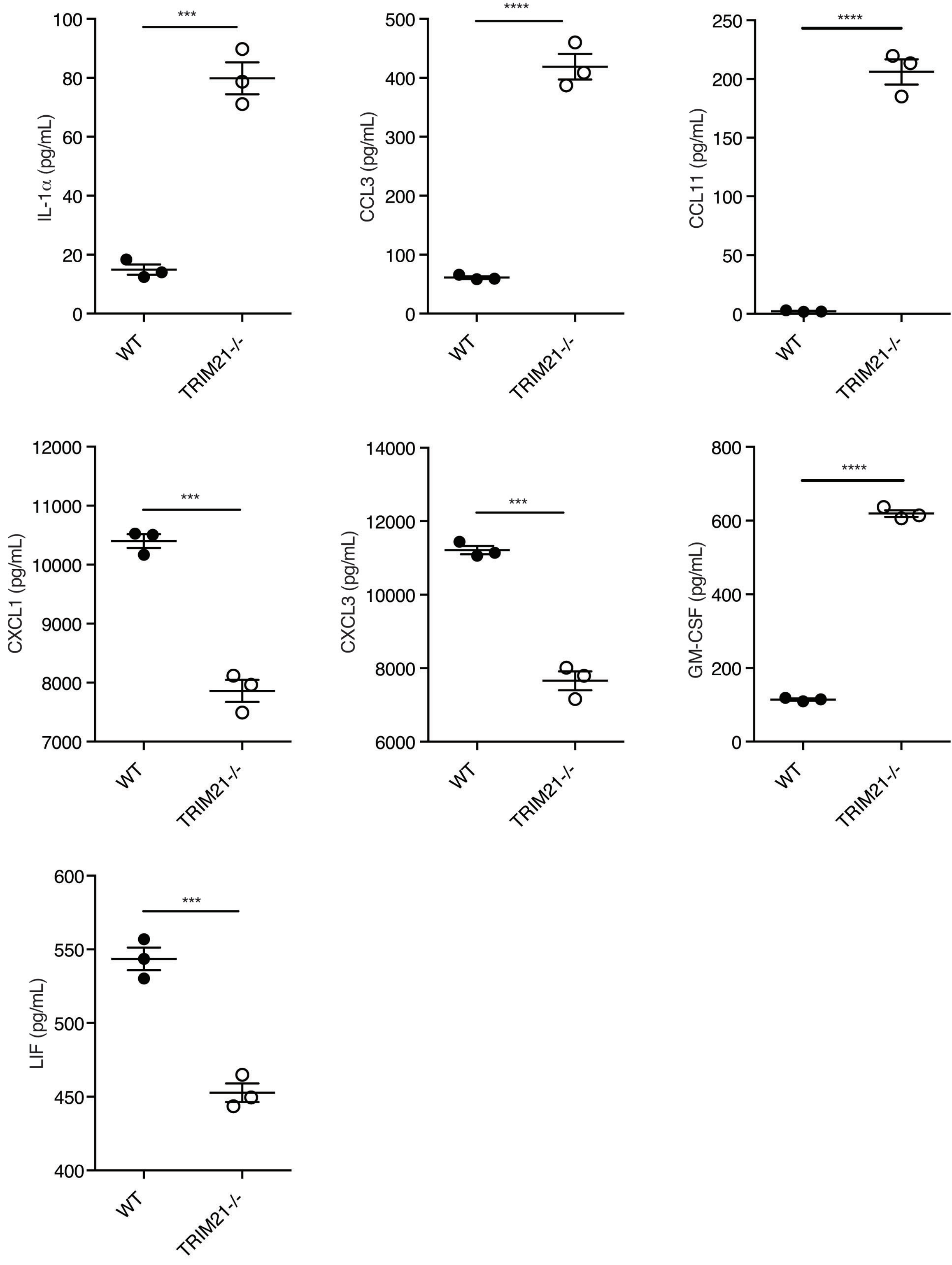

Supplement: Supplementary file 1 — Supplementary information [file 41598_2017_5487_MOESM1_ESM.pdf]
